# Supplementary material for: Integrative Genomic and Transcriptomic Profiling Reveals a Differential Molecular Signature in Uterine Leiomyoma versus Leiomyosarcoma
Source: Int J Mol Sci. 2022 Feb 16;23(4):2190. doi: 10.3390/ijms23042190 (PMC8877247; doi:10.3390/ijms23042190)
Supplement: Supplementary file 1 [file ijms-23-02190-s001.zip › ijms-1582000_Supplementary-Tables.pdf]

Supplementary Table S1. Pathogenic and likely pathogenic variants detected in the most frequently affected genes in leiomyosarcoma (LMS), based on HUGO Gene Nomenclature Committee and the Catalogue of Somatic Mutations in Cancer (HGVSc: HGVS coding sequence name; HGVSp: HGVS protein sequence name).

LM								
**Chromosome**	**Position**	**Reference**	**Alternative**	**Mutation**	**n_samples**	**SIFT**	**PolyPhen**	**COSMIC**
12	53012128	C	T	KRT73-c.181G>A	9	tolerated(0.17)	benign(0.01)	-
1	247024326	G	A	AHCTF1-c.4034C>T	8	tolerated(0.24)	benign(0.003)	https://cancer.sanger.ac.uk/cosmic/mutation/overview?id=103887737
22	40283427	A	G	ENTHD1-c.326T>C	8	deleterious(0.02)	probably_damaging(0.909)	https://cancer.sanger.ac.uk/cosmic/mutation/overview?id=102996182
16	684579	C	T	METTL26-c.565G>A	8	-	-	https://cancer.sanger.ac.uk/cosmic/mutation/overview?id=120485433
6	138491807	G	A	ARFGEF3-c.94G>A	7	tolerated(0.54)	-	https://cancer.sanger.ac.uk/cosmic/mutation/overview?id=101534468
5	118480316	G	A	DMXL1-c.2552G>A	7	tolerated(0.57)	-	-
21	46047779	G	A	KRTAP10-9-c.691G>A	7	-	-	-
1	150280621	T	G	MRPS21-c.223T>G	7	-	possibly_damaging(0.635)	https://cancer.sanger.ac.uk/cosmic/mutation/overview?id=163046164
13	111279817	A	G	NAXD-c.418A>G	7	tolerated(0.32)	benign(0.034)	https://cancer.sanger.ac.uk/cosmic/mutation/overview?id=102878237
5	140531700	A	G	PCDHB6-c.1862A>G	7	deleterious_low_confidence(0)	probably_damaging(0.962)	-
4	57797467	C	T	REST-c.2443C>T	7	tolerated_low_confidence(0.27)	benign(0.001)	-
6	4995464	C	T	RPP40-c.940G>A	7	deleterious(0)	probably_damaging(0.953)	https://cancer.sanger.ac.uk/cosmic/mutation/overview?id=114510553
**LMS**								
**Chromosome**	**Position**	**Reference**	**Alternative**	**Mutation**	**n_samples**	**SIFT**	**PolyPhen**	**COSMIC**
19	3982930	CG	GCC	EEF2-c.486_487delCGinsGGC	7	-	-	-

12	6646851	C	A	GAPDH-c.627C>A	7	-	-	-
22	32019810	T	A	PISD-c.58C>T	7	-	-	https://cancer.sanger.ac.uk/cosmic/mutation/overview?id=116284707
22	32019831	G	A	PISD-c.79A>T	7	-	-	-
2	9347277	A	T	ASAP2-c.44A>T	6	-	-	https://cancer.sanger.ac.uk/cosmic/mutation/overview?id=102543185
9	139262205	C	G	CARD9-c.1153G>C	6	-	-	-
18	52586545	A	G	CCDC68-c.746T>C	6	deleterious(0)	probably_damaging(0.962)	-
12	6646831	C	G	GAPDH-c.607C>G	6	-	-	-
12	6646832	T	C	GAPDH-c.608T>C	6	-	-	-
3	159482271	GGCA	G	IQCJ-SCHIP1-c.349_351delAGC	6	-	-	https://cancer.sanger.ac.uk/cosmic/mutation/overview?id=137467219
1	228456398	G	T	OBSCN-c.5581G>T	6	-	-	-
1	228456399	T	C	OBSCN-c.5582T>C	6	-	-	-
1	228456407	A	T	OBSCN-c.5590A>T	6	-	-	https://cancer.sanger.ac.uk/cosmic/mutation/overview?id=160852259

Supplementary Table S2 Most frequent copy number variants (CNVs) in the leiomyosarcoma (LMS) group (detected in at least 25% of LMS samples and in no leiomyoma samples).

Start	End	Chr	CNV type	Number of copies	Number of affected genes	Size	Frequency in LMS (%)
27467015	29412601	2	Deletion	1	33	1945586	29.41
214015545	215592774	2	Deletion	1	3	1577229	35.29
216003542	225338338	2	Deletion	1	86	9334796	32.35
29042365	32889966	13	Deletion	1	17	3847601	41.18
29470725	30947263	20	Duplication	3	28	1476538	32.35
134109653	135770995	9	Deletion	1	16	1661342	29.41
105206773	112327712	10	Deletion	1	24	7120939	29.41
112364698	123238463	10	Deletion	1	62	10873765	38.24
126524163	129896831	10	Deletion	1	16	3372668	29.41
129924556	135339987	10	Deletion	1	31	5415431	32.35
27494592	29482393	17	Duplication	3	26	1987801	29.41
56812191	59760035	17	Duplication	3	27	2947844	32.35

Supplementary Table S3. Quality control and metrics data obtained from whole-exome sequencing outcomes for leiomyoma (LM) and leiomyosarcoma (LMS) samples.

Sample name		Raw Bases gb	Aligned Bases gb	On target perc	Mean cov	gte10	Mean cov hem	gte100 hem
LM	8LM	19.6	12.8	75.8	145.4	98	840.3	99.8
	15LM	12.5	9.9	78.2	123	97.6	427.1	99.5
	16LM	19.7	15.5	79.6	194.6	98.3	708.2	99.9
	17LM	10	8.1	78	99.6	96.8	355.6	98.8
	18LM	15	9.7	85	132.2	97.5	495.1	99
	19LM	13.4	11	69.9	114.3	96.8	428.4	98.2
	21LM	13.8	9.3	79.5	114.6	97.4	443	99
	22LM	21.6	17.4	82.9	231.7	98.3	820.8	99.8
	23LM	14.5	11.2	77.9	136.4	98	474.2	99.7
	24LM	12.8	8.7	83.3	115.4	97.2	453.8	98.9
	25LM	26.2	20.2	83.3	269.1	98.5	966.2	99.9
	28LM	13.8	10.8	78.3	134.5	97.8	470	99.6
	30LM	14.8	11.4	78.2	140.3	98.2	490.7	99.7
	31LM	13.4	10.7	76.5	129.5	97.6	440	99.5
	32LM	27.9	22.5	82.2	296.7	98.4	1667	99.9
	34LM	19.9	15.1	81.1	195.5	98.3	676.3	99.8
	35LM	21.5	16.8	82	220.3	98.3	1191.9	99.9

	36LM	20.2	16	83.2	215.3	98.3	803.9	99.8
	41LM	23.6	18.2	82	238.8	98.3	1288.6	99.9
	44LM	21.5	14.6	85.3	198.5	98.1	757	99.7
	46LM	20.5	17.5	86.3	244.6	98.3	947.3	99.8
	47LM	21.5	14.5	83.6	194.1	98.1	766.6	99.5
	48LM	13.6	11.5	83.6	154.8	98.1	608.2	99.7
	49LM	12.8	8.8	83.7	118.6	97.6	467.6	99.1
	52LM	15.9	11.1	85.3	152.1	97.4	582	98.7
	54LM	17.8	12.1	84.9	162.9	98	694.2	99.4
	58LM	12.7	8.8	87.1	121.8	97.7	494	99.2
	59LM	21.9	14.2	82.9	187.5	98.1	719	99.6
	60LM	14.3	9.6	84.6	127.6	97.6	535.5	99.1
	61LM	11.1	7.7	84.9	102.7	97.3	415	98.7
	63LM	13.8	11.7	86.3	158.3	97.9	662.6	99.6
	65LM	11.6	10	84.6	135.8	98	557.8	99.6
	67LM	18.5	16.1	80.6	208.8	98.2	796.8	99.7
	68LM	18.3	15.7	86.1	217.6	98.2	891.8	99.8
	69LM	11.6	10	88.3	141.2	97.9	579.9	99.5
	71LM	12.8	11	87	151.1	97.8	645.3	99.5
	72LM	12.8	11	86.8	153.3	98.1	635	99.6
	73LM	13	11	73.1	128	97.9	531	99.6
	75LM	10.1	8.3	79.3	98.7	96.4	371.8	97.9
	76LM	13.1	11.1	75.7	132.7	97.9	550.9	99.5

	77LM	9.1	7.6	85.3	101.7	98.5	392.5	99.1
	78LM	11.2	9.4	84.6	124	97.1	459.6	98.7
	79LM	14.7	12.4	84.9	169.4	97.8	613	99.5
	80LM	19.3	13.6	70	138.8	97.6	589.9	99.3
**LMS**	LMS02	26.2	19.9	83.5	263.5	98.4	1558.2	99.9
	LMS03	38.2	19.6	79.3	236.6	98.3	1088.4	99.9
	LMS04	22.7	16.1	83.3	209.8	98.3	806.1	99.8
	LMS05	10.7	7.7	77	85.1	96.3	350.8	94.7
	LMS06	16.4	10	79.7	114.4	97.8	397.5	98.7
	LMS08	22.6	14	83.3	172	98.2	602.7	99.6
	LMS09	19.5	13.5	83.5	171.5	98.1	652.4	99.4
	LMS10	21.6	13.8	85.2	174.8	98.2	640.1	99.5
	LMS11	15.3	9.3	82.7	110.2	97.7	373.9	98.7
	LMS12	17.5	10.8	82.6	127.4	97.8	469.2	98.6
	LMS14	18	12.5	85.1	164	98.3	631.4	99.8
	LMS15	20.2	13.1	86.4	170.3	98.1	646	99.4
	LMS25	14.3	11.3	84.6	152.5	97.9	900.8	99.4
	LMS26	17.5	14.3	80.4	183.7	98	1021.6	99.8
	LMS31	21.5	14.8	83.6	192.2	98.2	703.1	99.7
	LMS32	14.9	10.5	83.4	134.6	98	517.6	99.5
	LMS33	22.7	16.4	83.8	217.6	98.4	808.5	99.8
	LMS34	8.4	6	76	66.6	95.8	251.8	95.1
	LMS35	12.5	8.3	84.7	109.8	96.7	408.3	97.9

	LMS36	19.8	13.5	82.8	178	97.6	643.9	99.2
	LMS39	12.9	8.4	84.8	110.8	97.6	465.3	99.2
	LMS48	11.7	8.1	84.9	107.8	97.4	463.1	98.7
	LMS49	24.2	16.1	66	157.4	98	612.9	99.4
	LMS50	11.9	8.5	85.5	113	97.4	482.3	99
	LMS52	10.5	8.5	81.4	106.7	97.6	420.3	99.3
	LMS53	13.3	8.9	79.8	110.8	97.4	453.7	98.9
	LMS54	19.1	13.4	84.8	172.4	97.9	646.7	99.6
	LMS55	11.3	7.7	85	101.2	96.8	425.2	97.3
	LMS56	12.1	8	81.6	102.1	97.3	421.4	98.8
	LMS57	24.2	20.4	84	279.6	98.2	966.5	99.5
	LMS58	12.7	11	81.4	144.2	98	574.4	99.5
	LMS60	13.9	11.3	71.7	128.4	97.8	507	99.4
	LMS61	12.4	10	85.3	124.8	97.3	550.2	97.9
	LMS62	11.7	9.5	87.2	129.8	97.7	547.7	99.3

Supplementary Table S4. Primer sequences and fragment sizes from six mononucleotide repeat markers tested for microsatellite instability. In each case, the antisense primer was labeled with a fluorescent dye. D3S1260, a highly polymorphic dinucleotide repeat marker, was used as an internal control.

Gene	Primer	Sequence	Primer length (nt)	Fluorescent 5' label	Fragment size (pb)
BAT25	BAT25_FW	TCGCCTCCAAGAATGTAAGT	20	VIC	122–124
	BAT25_RV	TCTGCATTTTAACTATGGCTC	21	-	
BAT26	BAT26_FW	TGACTACTTTTGACTTCAGCC	21	6FAM	116–117
	BAT26_RV	AACCATTCAACATTTTTAACCC	22	-	
BAT40	BAT40_FW	AGTCCATTTTATATCCTCAAGC	22	PET	142–146
	BAT40_RV	GTAGAGCAAGACCACCTTG	19	-	
NR21	NR21_FW	TAAATGTATGTCTCCCCTGG	20	VIC	98–99
	NR21_RV	ATTCCTACTCCGCATTCACA	20	-	
NR22	NR22_FW	GAGGCTTGTCAAGGACATAA	20	6FAM	138–140
	NR22_RV	AATTCGGATGCCATCCAGTT	20	-	
NR27	NR27_FW	AACCATGCTTGCAAACCACT	20	6FAM	86–87
	NR27_RV	CGATAATACTAGCAATGACC	20	-	
D3S1260	D3S1260_FW	CTACCAGGGAAGCACTGTAG	20	6FAM	171–189
	D3S1260_RV	CATGTACCTGAGCACCTACTG	21	-	

Supplementary Table S5. Sequencing reads aligned reads per sample, and percentage of aligned reads from RNAseq data for leiomyoma (LM) and leiomyosarcoma (LMS) samples.

Sample		Number of reads	Number of aligned reads	% of aligned reads
LM	8LM	53.300.426	46.541.932	87.32
	15LM	65.529.986	56.978.323	86.95
	16LM	85.795.012	75.602.565	88.12
	17LM	69.618.698	60.902.437	87.48
	18LM	74.612.844	65.607.074	87.93
	19LM	57.662.702	50.345.305	87.31
	21LM	65.951.562	57.760.378	87.58
	22LM	82.063.226	71.690.434	87.36
	23LM	30.637.470	26.881.316	87.74
	24LM	72.902.072	62.214.628	85.34
	25LM	56.371.092	48.856.825	86.67
	28LM	31.515.306	28.108.501	89.19
	30LM	25.013.202	22.654.457	90.57
	31LM	34.871.992	30.872.175	88.53
	32LM	69.500.458	59.325.591	85.36

	34LM	73.270.816	61.364.308	83.75
	35LM	82.184.314	70.810.005	86.16
	36LM	59.771.062	51.976.916	86.96
	41LM	51.180.652	45.136.217	88.19
	44LM	39.804.674	21.327.344	53.58
	46LM	61.842.240	54.377.882	87.93
	47LM	74.899.898	66.009.280	88.13
	48LM	54.597.600	30.268.909	55.44
	49LM	80.485.526	71.583.827	88.94
	52LM	76.145.418	66.543.481	87.39
	54LM	42.774.568	23.859.654	55.78
	58LM	42.379.782	22.465.522	53.01
	59LM	49.091.420	41.439.295	84.41
	60LM	72.095.082	62.583.938	86.81
	61LM	67.285.822	41.427.881	61.57
	63LM	66.928.162	43.750.939	65.37
	65LM	65.902.554	40.279.641	61.12
	67LM	61.125.038	33.083.927	54.13
	68LM	55.827.354	29.349.836	52.57

	69LM	45.628.616	29.674.570	65.04
	71LM	77.011.662	65.226.952	84.70
	72LM	80.215.932	69.039.847	86.07
	73LM	80.904.648	69.345.396	85.71
	75LM	78.473.002	67.033.600	85.42
	76LM	40.300.066	32.986.612	81.85
	77LM	71.785.384	62.063.848	86.46
	78LM	66.605.266	54.956.005	82.51
	79LM	78.608.486	60.267.161	76.67
	80LM	56.010.968	31.296.128	55.88
**LMS**	LMS02	41.750.886	36.415.123	87.22
	LMS03	142.681.492	90.092.661	63.14
	LMS04	32.354.842	26.666.861	82.42
	LMS05	56.453.814	40.291.087	71.37
	LMS08	57.906.670	39.567.628	68.33
	LMS10	68.118.260	49.848.943	73.18
	LMS11	57.647.630	36.358.360	63.07
	LMS12	49.080.458	32.132.976	65.47
	LMS13	71.048.488	49.513.691	69.69

	LMS14	69.820.774	48.478.309	69.43
	LMS15	58.169.588	37.827.683	65.03
	LMS25	84.796.512	71.949.840	84.85
	LMS26	89.645.476	73.993.376	82.54
	LMS31	65.520.994	55.784.574	85.14
	LMS32	24.717.496	22.122.159	89.50
	LMS33	38.324.476	32.035.429	83.59
	LMS34	25.328.238	21.103.488	83.32
	LMS35	67.985.942	50.329.993	74.03
	LMS36	39.985.408	33.867.641	84.70
	LMS38	79.790.396	50.499.342	63.29
	LMS39	41.089.440	25.453.881	61.95
	LMS42	53.981.420	36.320.049	67.28
	LMS43	56.251.340	36.568.996	65.01
	LMS44	55.805.988	35.874.879	64.29
	LMS48	59.323.790	42.585.583	71.79
	LMS49	31.311.200	23.095.141	73.76
	LMS52	33.244.482	27.934.507	84.03
	LMS53	55.294.234	45.755.979	82.75

	LMS55	67.359.888	59.064.518	87.69
	LMS56	74.670.280	63.654.547	85.25
	LMS57	41.339.950	35.229.905	85.22
	LMS60	75.111.574	65.884.117	87.72
	LMS61	67.587.936	58.056.347	85.90
	LMS62	65.927.122	56.085.851	85.07

Supplementary Table S6. Primers for RT-qPCR validation, including the most differentially expressed genes identified from RNAseq data.

Gene	Primer	Sequence	Primer length (nt)	GC content (%)	Tm (ºC)	Amplicon size (nt)
SPAG5	SPAG5_FW	AGTGCTTTCACCCGAGTAGC	20	55	60.04	163
	SPAG5_RV	TTCGCTGCAGAGTCCTGATG	20	55	60.11	163
NUF2	NUF2_FW	AACAGTTAAACGCCGCACAC	20	50	59.97	168
	NUF2_RV	TTCCCTCTTGCAGCACTATCG	21	52.38	60.13	168
BUB1B	BUB1B_FW	TGGGTCCTTCTGGAAACTTAGC	22	50	59.96	100
	BUB1B_RV	GGCCTCATCATTGGCATTCAG	21	52.38	59.66	100
KIF14	KIF14_FW	TTTTGCAGGGATGCTGTTTGG	21	47.62	59.93	109
	KIF14_RV	AGAACTGCTAACTGCCCCAC	20	55	59.96	109
FANCI	FANCI_FW	GAGCACTCCTGAGAGCCATC	20	60	59.90	111
	FANCI_RV	AATCCCCCGATTCCACCAAC	20	55	60.03	111
ACTB	ACTB_FW	CACACTGTGCCCATCTACGA	20	55	61	199
	ACTB_RV	TAGCTCTTCTCCAGGGAGGA	20	55	60.5	199
