# Supplementary material for: Integrative Genomic and Transcriptomic Profiling Reveals a Differential Molecular Signature in Uterine Leiomyoma versus Leiomyosarcoma
Source: Int J Mol Sci. 2022 Feb 16;23(4):2190. doi: 10.3390/ijms23042190 (PMC8877247; doi:10.3390/ijms23042190)
Supplement: Supplementary file 1 [file ijms-23-02190-s001.zip › suppM&M_IJMS_01172022_rev_Integrative_LMS-LM.pdf]

Supplementary material and methods

Nucleic acid isolation, quality control and quantification

DNA and RNA from 5–10-μm-thick FFPE tumor sections were isolated using GeneRead DNA FFPE kit and miRNAeasy FFPE kit (Qiagen) following manufacturer instructions. Quality control and quantification analysis were performed using Qubit 2.0 Fluorometer (Thermo Scientific, Waltham, MA, USA) and 2100 Bioanalyzer system (Agilent, Santa Clara, CA, USA). Additionally, we measured amplification potential of nucleic acids by assessing ΔCq value through quantitative PCR, after normalization for a fixed input mass. Only DNA samples with ΔCq ≤ 5 and 1 μg total DNA were included. Consequently, five out of 39 LMS and five out 49 LM cases in the experimental cohort were excluded for whole exome sequencing due to insufficient quantity and/or quality of DNA. RNA samples with 100 ng total RNA and DV200 ≥30% (percentage of RNA fragments >200 nucleotides in length) were selected for further experiments. Accordingly, five out of 39 LMS and five out of 49 LM cases in the experimental cohort were excluded for RNAseq due to insufficient quantity and/or quality of RNA.

DNA library preparation, targeted exome sequencing, and mapping

DNA sequencing libraries from 44 LM and 34 LMS were constructed using the KAPA Hyper Prep kit (Roche, Switzerland). All exons were captured by a custom-designed SeqCap EZ MedExome kit (NimbleGen; Roche, Switzerland), which targeted exons and neighboring introns of 571 hematological-associated genes. Lastly, each library was loaded in a HiSeqXTen platform (Illumina, USA).

DNA sequence data were demultiplexed and formatted in FASTQ files (FASTQ 1.9 files with Phred+33) containing at least 32 million DNA reads for each sample. Reads were aligned to the human hg19 genome (CRGCh37) using the Burrows-Wheeler Alignment tool software (v0.7.17), with -M and -R options to mark short and split alignments as secondary and add read group information, respectively (https://github.com/lh3/bwa).

SAM files were then converted to coordinate sorted BAM files using samtools v1.9 (https://github.com/samtools/samtools), and Picard Tools v2.20.1 (https://github.com/broadinstitute/picard) was used to mark and remove duplicates. Interval reference files (design files) for the SeqCap EZ MedExome were also provided. Specifically, ±100 bases were added to the capture bed file for calling with a mean depth higher than 150X, with >80% of target regions covered.

Small variants, including somatic SNVs and indels, were identified from LM and LMS tumors using Freebayes (v1.3.2) with base quality ≥3 and alternate fraction ≥0.12 (https://github.com/freebayes/freebayes). Accordingly, for variants with quality <20, homopolymer regions and missing values were filtered out with BCFtools (v1.9) (https://github.com/samtools/bcftools). Variants with >5% frequency in gnomAD (https://gnomad.broadinstitute.org/) or with a non-PASS filter in BRAVO (https://www.nhlbiwgs.org/) were removed. SnpEff (v4.3t) was used for variant annotation (https://github.com/pcingola/SnpEff).

Analysis of somatic mutational signatures inferred from SNVs was done using MutationalPatterns (https://github.com/UMCUGenetics/MutationalPatterns). The profile of each signature was displayed using the six substitution subtypes and determined based on the 96-base substitution model.

To evaluate MSI, tumoral DNA was amplified for of six mononucleotide repeat markers: BAT25, BAT26, BAT40, NR21, NR22, and NR27, as well as D3S1260 as an internal control, after which amplified fluorescent PCR products were run on ABI 3730XL DNA Analyzer (Applied Biosystems) and analyzed using Gene Mapper.

RNA library preparation, sequencing, and mapping

RNAseq data were demultiplexed to generate FASTQ files containing ≥20 million RNA reads per sample. Reads were aligned to the human hg19 genome using STAR (https://github.com/alexdobin/STAR). After quality filtering, we obtained an average of 47 million uniquely mapped reads per sample. Finally, gene transcript abundance was estimated using HTseq (https://github.com/simon-anders/htseq).

Detection of chromosomal rearrangements and validation

Based on RNAseq data, we obtained a list of candidate fusions using the CRCh37 reference genome and default parameters, using. only fusions classified as high confidence and with at least five total split reads. Of the detected fusions, ATRX and RAD51B rearrangements were validated by immunohistochemistry. 5 µm FFPE tissue sections were assessed using a 1:10 and 1:100 dilution of mouse monoclonal antibodies (Thermo Fisher Scientific). Negative controls omitted the primary antibody from the diluents.

Differential expression analysis, validation by qRT-PCR and functional analysis

Trimmed mean of M-values method was used to compute normalization factors, and tagwise dispersions were calculated and subjected to a quasi-likelihood F-test.

Validation was performed in duplicate using the Sybr Fast qPCR Kit (KAPA Biosystems Inc., Roche), with specific amplifying primer pairs. PCR products were analyzed using the comparative Ct method (2−ΔΔCT) and normalized to the housekeeping gene *β-actin*, showing values as relative to the mean in the control group.

Finally, to identify enriched biological functions and pathways, we performed KEGG and Reactome pathway enrichment analysis using ClusterProfiler (https://github.com/YuLab-SMU/clusterProfiler). Additionally, GO terms with $p < 0.01$ were summarized using REVIGO.

Gene selection for classification model and validation

To build and train the models, we reviewed previously identified DEGs. Genes with variances close or equal to 0 or with very high correlation values between them were removed. CPM values were pre-processed using caret, which standardized them to minimize the influence of different scales and variances in the final model by centering and scaling the data (mean = 0, SD = 1, respectively).

AdaBoost models were trained with 10-fold stratified cross-validation to obtain robust estimates of tumor classification capabilities, following a two-step approach. In the first step, five subsets of samples were created from the training data. From them, four subsets were used for fitting the model, and the other subset was used for feature pruning. Since model composition varied each time due to the probabilistic nature of classification models, fitting and feature pruning were repeated 10 times for each feature pruning subset, generating a total of 50 models.

In the second step, these models were combined to generate a single aggregate AdaBoost model, which contained the best average performance across all pruning subsets and included a total of 19 relevant genes.

For model validation, re-sequencing of all LM (n = 44) and LMS (n = 34) samples was performed, also adding a new set of 8 LM and 10 LMS samples. Sequencing data from the Illumina NextSeq500 sequencer were demultiplexed and aligned to the CanTRAN hg19 reference genome using BWA mem (https://github.com/lh3/bwa). Coverage for each of the 19 target genes was calculated using bedtools (https://github.com/arq5x/bedtools2) and normalized based on total reads per sample.
